# Mutation Profiling in Cholangiocarcinoma: Prognostic and Therapeutic Implications

**DOI:** 10.1371/journal.pone.0115383

**Published:** 2014-12-23

**Authors:** Chaitanya R. Churi, Rachna Shroff, Ying Wang, Asif Rashid, HyunSeon C. Kang, Jacqueline Weatherly, Mingxin Zuo, Ralph Zinner, David Hong, Funda Meric-Bernstam, Filip Janku, Christopher H. Crane, Lopa Mishra, Jean-Nicholas Vauthey, Robert A. Wolff, Gordon Mills, Milind Javle

**Affiliations:** The University of Texas M.D. Anderson Cancer Center, Houston, Texas, United States of America; Baylor College of Medicine, United States of America

## Abstract

**Background:**

Cholangiocarcinoma (CCA) is clinically heterogeneous; intra and extrahepatic CCA have diverse clinical presentations. Next generation sequencing (NGS) technology may identify the genetic differences between these entities and identify molecular subgroups for targeted therapeutics.

**Methods:**

We describe successful NGS-based testing of 75 CCA patients along with the prognostic and therapeutic implications of findings. Mutation profiling was performed using either a) NGS panel of hotspot regions in 46 cancer-related genes using a 318-chip on Ion PGM Sequencer or b) Illumina HiSeq 2000 sequencing platform for 3,769 exons of 236 cancer-related genes plus 47 introns from 19 genes to an average depth of 1000X. Clinical data was abstracted and correlated with clinical outcome. Patients with targetable mutations were referred to appropriate clinical trials.

**Results:**

There were significant differences between intrahepatic (n = 55) and extrahepatic CCA (n = 20) in regard to the nature and frequency of the genetic aberrations (GAs). *IDH1* and DNA repair gene alterations occurred more frequently in intrahepatic CCA, while *ERBB2* GAs occurred in the extrahepatic group. Commonly occurring GAs in intrahepatic CCA were *TP53* (35%), *KRAS* (24%), *ARID1A* (20%), *IDH1* (18%), *MCL1* (16%) and *PBRM1* (11%). Most frequent GAs in extrahepatic CCA (n = 20) were *TP53* (45%), *KRAS* (40%), *ERBB2* (25%), *SMAD4* (25%), *FBXW7* (15%) and *CDKN2A* (15%). In intrahepatic CCA, *KRAS*, *TP53* or *MAPK/mTOR* GAs were significantly associated with a worse prognosis while *FGFR* GAs correlated with a relatively indolent disease course. *IDH1* GAs did not have any prognostic significance. GAs in the chromatin modulating genes, *BAP1* and *PBRM1* were associated with bone metastases and worse survival in extrahepatic CCA. Radiologic responses and clinical benefit was noted with EGFR, FGFR, C-met, B-RAF and MEK inhibitors.

**Conclusion:**

There are significant genetic differences between intra and extrahepatic CCA. NGS can potentially identify disease subsets with distinct prognostic and therapeutic implications.

## Introduction

CCA represents the second most common primary liver cancer worldwide. Several recent epidemiological reports indicate that its incidence and mortality is rising, particularly in the western world [1]–[6]. The overall survival of this disease remains dismal due to delayed detection, suboptimal response to standard therapy and underlying liver disease such as non-alcoholic steatohepatitis (NASH), which can limit liver-directed therapies [7], [8]. The current clinical classification of CCA is based on its anatomic location and includes the intrahepatic, hilar and distal subgroups. The Liver Cancer Study Group of Japan has described three morphological variants of intrahepatic CCA; these include the mass-forming, intraductal and peri-hilar types, based on the patterns of disease spread [9]. These classifications have improved the understanding and surgical management of this disease. The location of the tumor (intra vs. extrahepatic) has no therapeutic implications currently in the advanced disease setting as both types receive gemcitabine-based chemotherapy. Their clinical course can vary; hilar CCA is associated with prolonged survival even in the locally advanced disease setting with liver transplantation, while the same modality in intrahepatic CCA has suboptimal results. Distal extrahepatic CCA has a clinical course that is similar to pancreatic adenocarcinoma. Underlying genetic differences between these two entities have not yet been adequately explored.

Recently, there have been several reports in regards to genomic profiling of intrahepatic CCA. Ong *et al.*, conducted a whole exome sequencing study of patients with Opisthorchis viverrini-related CCA from East Asia. Using Sanger sequencing, they noted activating mutations in *P53, KRAS, SMAD4* and 10 other newly implicated genes including *MLL3, ROBO2, RNF3* and *PEG3* [10]. This group further compared the genomic profile of O. viverrini-related and non-O. viverrini-related CCAs and demonstrated statistically significant differences in mutation patterns between these entities. Jiao et al. from Johns Hopkins, performed an exome sequencing analysis of 32 cases of intrahepatic CCA and demonstrated the relatively common occurrence of GAs in chromatin regulation genes [11]. Similar studies in extrahepatic CCA are lacking. Furthermore, the clinical phenotype or prognosis associated with these GAs and the effect of targeted therapies in the various CCA subsets remains to be demonstrated.

## Materials and Methods

### Case Selection

Surgically resected or tumor core biopsy FFPE specimens were obtained for 75 patients with CCA. All patients selected were those with pathologically confirmed CCA and a minimum of 3 months of follow-up. Patients signed an informed consent that covered review of medical records, and studies for correlated research. The study was approved by the Institutional Review Board of MD Anderson Cancer Center. Patient demographics, clinical data, survival data and treatment history were retrieved from medical records. For these cases, if adequate material was procured, somatic mutation analysis was first performed using a NGS panel of hotspot regions in 46 cancer-related genes described below. In cases where targetable mutations were not identifiable using this technology or if patients were being screened for clinical trials that required a wider NGS panel, Foundation Medicine (Boston, MA) performed additional sequencing with the IIlumina Hiseq2000 Platform.

### Tumor samples

The paraffin embedded blocks were sectioned, and hematoxylin & eosin (H&E) stained slides were reviewed by surgical pathology to confirm the tumor content in each section. Ten serial sections (4 µm) were cut from selected tissue blocks and areas with tumor tissue were macrodissected from those slides using the H&E slides as templates. Clinical data were abstracted from all the patients. Approval for the study was obtained from the institutional review board at MD Anderson Cancer Center.

### DNA Extraction

The pathologic diagnosis of each case of CCA was confirmed on routine H & E slides. All samples sent for DNA extraction contained a minimum of 20% DNA derived from tumor cells. DNA was extracted from 40 mm of FFPE tissue using the Maxwell 16 FFPE Plus LEV DNA Purification kit (Promega) and quantified using a standardized PicoGreen fluorescence assay (Invitrogen).

### Next Generation Sequencing

An amplicon library was generated from 10 ng of DNA from each sample using the Ion Ampliseq Cancer Panel (Life Technologies). Formalin-fixed, paraffin-embedded cell pellets of the H2122 cell line diluted in the HL60 cell line were used as control. The 46 genes in the panel for detection of targetable mutations included: *AKT1, BRAF, FGFR1, GNAS, IDH1, FGFR2, KRAS, NRAS, PIK3CA, MET, RET, EGFR, JAK2, MPL, PDGFRA, PTEN, TP53, FGFR3, FLT3, KIT, ERBB2, ABL1, HNF1A, HRAS, ATM, RB1, CDH1, SMAD4, STK11, ALK, SRC, SMARCB1, VHL, MLH1, CTNNB1, KDR, FBXW7, APC, CSF1R, NPM1, SMO, ERBB4, CDKN2A, NOTCH1, JAK3, and PTPN11*. Primers for PCR amplification included the 190-primer pair pool provided by the vendor with an additional primer pair that was custom added to cover the ‘hotspot’ location on codon 17 of AKT1. Following PCR amplification of target sequences, barcodes were ligated to the amplicons using the Ion Xpress Barcode Adaptors Kit (Life Technologies). Library quantification was then performed using the Bioanalyzer High Sensitivity DNA Chip (Agilent Technologies, Santa Clara, CA). The library was diluted in nuclease-free water to obtain a final concentration of 16 pM. Emulsion PCR was performed manually using the Ion Xpress Template Kit (Life Technologies) followed by manual breaking of the emulsion to isolate the ion spheres (ISPs). The quality of the DNA following PCR was measured using the Qubit IonSphere Quality control kit (Life Technologies). Selective ISPs with DNA were isolated and sequenced on a Ion 316 Chip (4 samples/chip) or a Ion 318 Chip (8 samples/chip) using the vendor-provided sequencing kit (Life Technologies). Successful sequencing of a sample required at least 300 000 reads with a quality score of AQ20 (1 misaligned base per 100 bases). For a wild-type call, a minimum coverage of 250× was required. As tumor specimens were admixed with normal tissue, a minimum coverage of 500× with at least 10% frequency was used as cutoff for a variant to be considered true. All variants detected by Ion PGM with at least 10% frequency were selected for confirmation by alternate platforms. Further details regarding the methodology and analysis have been described previously [12]. NGS performed by Foundation Medicine used the Illumina Hiseq 2000 Platform for 236 targetable GA's and the methods for the same have been described previously [13], [14].

### Immunohistochemistry for HER2/Neu

A standard technique for fixing tissue in formalin and embedding it in paraffin was used. The 4-µm-thick histologic sections obtained from the TMAs were deparaffinized and hydrated in decreasing alcohol concentrations. Antigens were recovered by exposure to microwaves in citrate buffer (pH 6.0) and washed in PBS (pH 7.4). The monoclonal antibody anti-ErbB2 (NCL-CB11; Novocastra) was used at a dilution of 1∶40. The primary antibody was incubated at room temperature for 60 minutes and then incubated with the complex Super Picture Polymer Detection Kit (Zymed) in a Dako autostainer. Standard criteria for HER2/Neu scoring were utilized [15].

### Statistical Analysis

We investigated potential associations between genetic mutations and survival in this cohort. All patients who had at least 3 months of follow up were included for the survival analysis. Overall survival (OS) was the chosen primary endpoint for this analysis. Progression-free survival (PFS) was a secondary endpoint as in prior clinical trials in CCA, PFS correlated accurately with overall survival (OS) [16]. Patient characteristics, age, gender, primary tumor site (intra- and extrahepatic), treatments administered, GA's observed, disease course, progression date and current status were recorded. The PFS and OS were estimated by using the Kaplan–Meier method. For survival analysis, the log rank test was used for discrete variables and the Cox proportional hazard model was used for continuous variables. All variables that were significant in univariate analysis and variables of our interest were entered into a multivariate model. The Cox proportional hazards model was used to calculate hazard ratio and 95% confidence intervals. A *p*-value of less than 0.05 was considered significant.

### Ingenuity Pathway Analysis

The web-based pathways analysis tool IPA (Ingenuity Systems, www.ingenuity.com) was used to identify signaling pathways affected in CCA. This web-based entry tool allows for the mapping of gene expression data into relevant pathways based on their functional annotation and known molecular interactions. This knowledge coming from published, peer-reviewed scientific publications is stored in the Ingenuity Pathways Knowledge Base (IPKB), and is continuously updated. The mutated genes were uploaded into IPA. Canonical pathways analysis identified molecular pathways from the IPA library of canonical pathways (part of the IPKB) that were most significant to the data set. Genes from the data set that were associated with a canonical pathway in the IPKB were considered for the analysis. The significance of the association between the genes from the dataset and the canonical pathway (in the IPKB) was measured. Fisher's exact test was used to calculate a *p-*value determining the probability that there is an association between the genes in the dataset and the canonical pathway that cannot be explained by chance alone.

## Results

**Figure 1:**
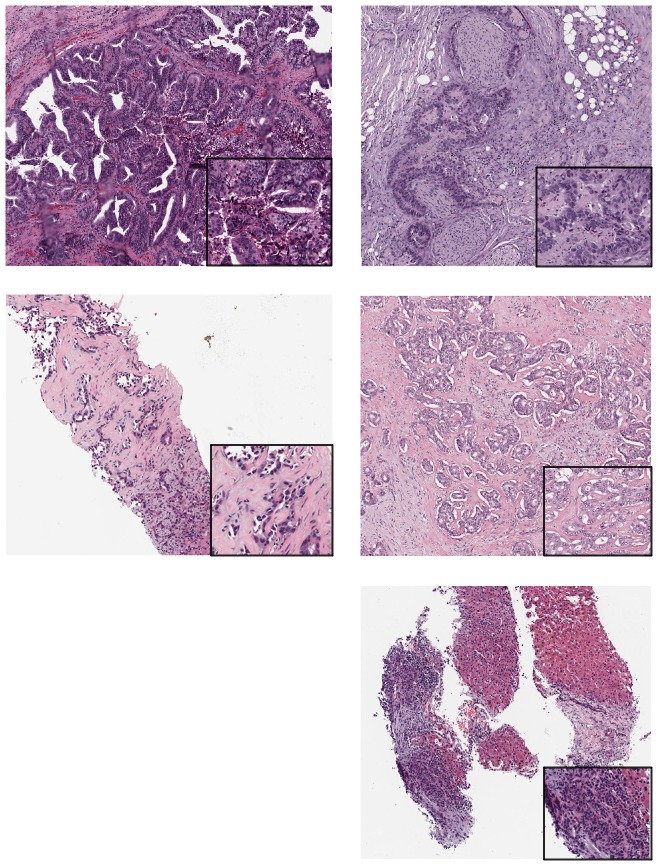
Representative histology of select tumors with the respective GA (40x, 200x). [A. *ERBB2* (S310F) B. *ERBB2* (S310F) C. *BAP1* (C91*) D. FGFR2-KIAA1598 fusion E. FGFR2-NOL4 fusion].

**Figure 2:**
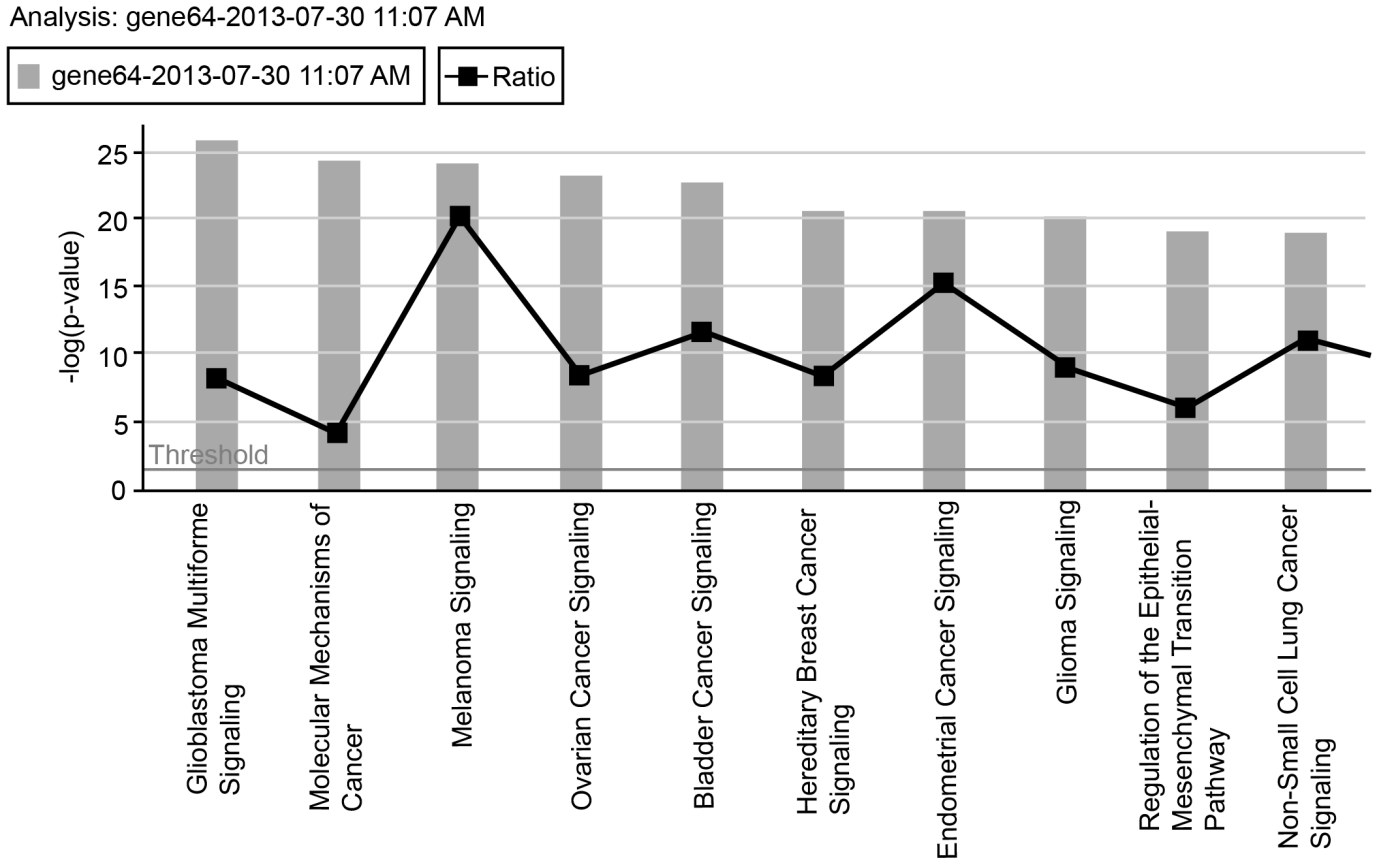
Ingenuity Pathway Analysis indicates canonical signaling pathways involved in CCA. Homology noted with more commonly occurring solid tumors including melanoma and glioblastoma.

Seventy-five cases of CCA were analyzed for GAs using NGS. Tissue for NGS was obtained from the following sites: 26 samples were from surgical resections and 49 were from biopsies. Forty biopsy specimens were from the liver, 2 from retroperitoneal and peripancreatic lymph nodes, 3 from omentum nodules and 4 from peritoneum nodules. The median follow-up duration in this study was 19 months. Majority of the cases (90%) were investigated using the Illumina Hiseq Platform. Out of the 75 cases, 55 were intrahepatic CCAs and 20 were extrahepatic CCAs. For the purpose of our analysis, we considered these two entities separately. There were notable genetic differences between intra and extrahepatic CCA. *IDH1* mutations occurred exclusively in intrahepatic CCA while *ERBB2* mutations were seen in extrahepatic CCA. Other genetic differences between these two entities are depicted in Table 1. The allele frequency and copy number of these GAs are depicted in S1 Table. There were no morphological differences in the respective tumors associated with these GAs on light microscopy (Fig. 1). IPA canonical pathways representing these cases are represented in Fig. 2. There is close homology between the canonical pathways noted in CCA with those seen in glioblastoma, melanoma, hereditary breast cancer and bladder cancer. The affected molecular and cell functions identified on the IPA were cell cycle regulation, cellular growth, death, DNA replication and repair. We analyzed the frequency and prognostic significance of the GAs separately in intra and extrahepatic CCA.

**Table 1:** Genetic differences identified between Intrahepatic and Extrahepatic CCA

GENETIC ABNORMALITY	INTRAHEPATIC CCA (N = 55)	EXTRAHEPATIC CCA (N = 20)	ODDS RATIO	p-value	95% CI
	N(%)	N(%)			
GENES					
*TP53*	16 (29.1%)	9 (45%)	0.506	0.268	0.154–1.669
*KRAS*	13 (23.6%)	8 (40%)	0.469	0.244	0.138–1.626
*ARID1A*	11 (20%)	1 (5%)	4.678	0.164	0.599–214.83
*ERBB2*	1 (1.8%)	5 (20%)	0.058	0.004	0.001–0.576
*PBRM1*	6 (10.9%)	1 (5%)	2.305	0.667	0.252–112.51
*BAP1*	5 (9.1%)	2 (10%)	0.901	1	0.132–10.26
*FBXW7*	3 (5.5%)	3 (15%)	0.333	0.333	0.041–2.719
*SMAD4*	2 (3.6%)	5 (25%)	0.333	0.333	0.010–0.804
*IDH*	13 (23.6%)	0 (0%)	Infinite	0.01	1.274 – Inf
PATHWAYS					
MAP-ERK	19 (34.5%)	11 (55%)	0.437	0.121	0.133–1.389
mTOR	14 (25.5%)	8 (40%)	0.517	0.258	0.154–1.776
DNA Repair	9 (16.4%)	8 (40%)	0.299	0.058	0.081–1.094
FGF Pathway	7 (12.7%)	1 (5%)	2.741	0.673	0.316–131.27
Chromatin Modification	18 (32.7%)	3 (15%)	2.724	0.157	0.659–16.364

### Intrahepatic CCA

Median age of the 55 patients with intrahepatic CCA was 60 years (range 24–84). Patient demographics are described in Table 2. The median PFS for these patients was 6.1 months and median OS was 10.9 months.

**Table 2:** Patient demographics and tumor characteristics.

CHARACTERISTIC	INTRAHEPATIC (N = 55)	EXTRAHEPATIC (N = 20)
Male	16 (29%)	13 (65%)
Female	39 (71%)	7 (35%)
Asian	2 (4%)	1 (5%)
Hispanic	8 (15%)	0 (0%)
Black	3 (5%)	0 (0%)
White	42 (76%)	19 (95%)
Poorly differentiated	28 (47%)	7 (35%)
Moderate differentiated	26 (48%)	11 (55%)
Well differentiated	1 (2%)	0 (0%)
Grade N/A	0 (0%)	2 (10%)
AJCC II	4 (7%)	6 (30%)
AJCC III	17 (31%)	5 (25%)
AJCC IV	34 (62%)	9 (45%)
Surgery without adjuvant therapy	7 (13%)	7 (35%)
Radiation therapy	10 (18%)	1 (5%)
Surgery & adjuvant chemoradiation	6 (11%)	7 (35%)
Systemic Therapy	32 (58%)	5 (25%)

**Figure 3:**
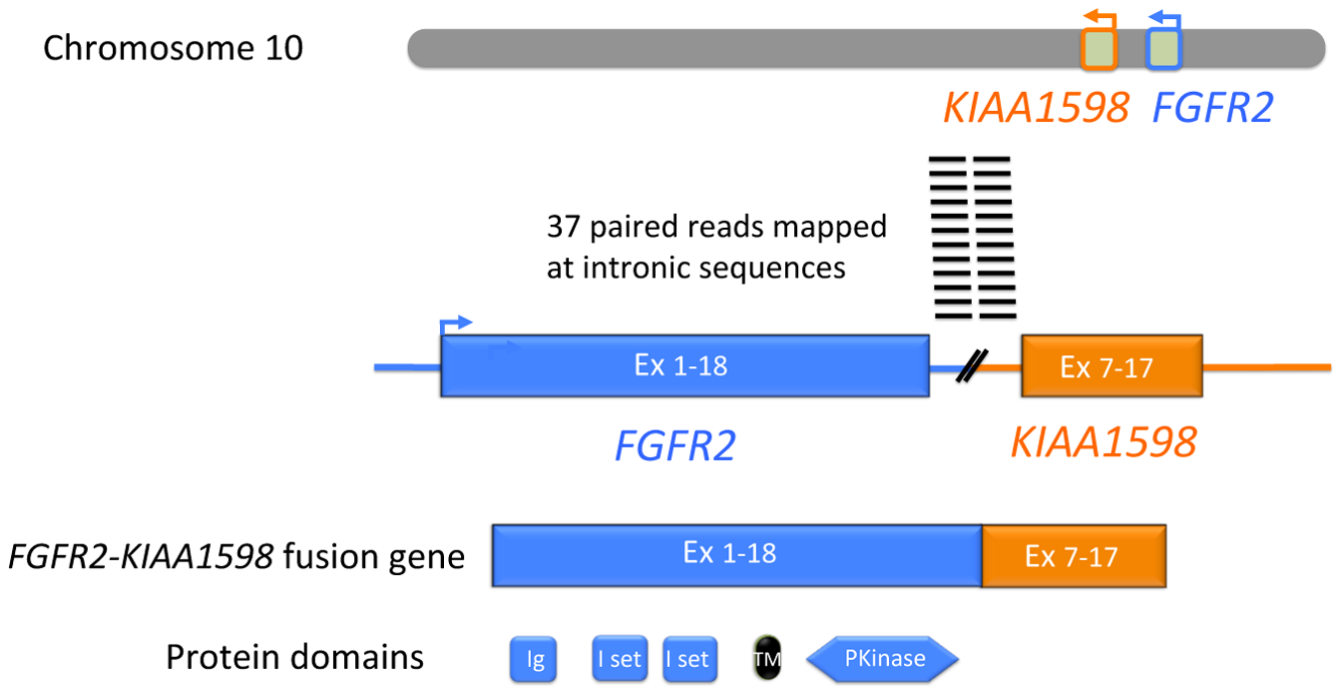
Schematic of *FGFR2-KIAA1598* fusion gene. These fusions have been proven oncogenic and are potentially susceptible to FGFR inhibitors.

162 GAs were identified from 55 intrahepatic CCA patient samples with an average of 2.95 GAs/patient (range 0–7). GAs identified were mutations (66%), amplifications (20%), loss/deletions (7%) and others (7%). Only 4 (7%) tumors showed no detectable GAs using the NGS. Most frequent GAs were *TP53* (35%), *KRAS* (24%), *ARID1A* (20%), *IDH1* (18%), MCL1 (16%) and *PBRM1* (11%). FGF pathway GAs occurred in 13% of cases, these included mutations, amplifications and *FGFR* fusion genes. The latter were noted in 3 cases: *FGFR2-KIAA1598, FGFR2-NOL4* and *FGFR2-PARK2* fusions. A schematic representation of the *FGFR2-KIAA1598* fusion gene is depicted in Fig. 3. Signaling pathways associated with these mutations are depicted in Table 3. MAPK, chromatin modification and mTOR pathway aberrations were relatively common (35%, 33% and 25%, respectively) followed by DNA repair (16%) and FGF signaling (13%).

**Table 3:** Classification of select genomic variations identified based on roles in cell signaling pathways.

PATHWAY	GENES	INTRAHEPATIC	EXTRAHEPATIC
FGF	*FGF19, FGF3, FGFR2, FGF4, FGFR3, FGFR2-KIAA1598* fusion, *FGFR2-NOL4* fusion, *FGFR2-PARK2* fusion.	13%	5%
mTOR	*FBXW7, PIK3CA, PTEN, NF1, NF2, PIK3R1, STK11, TSC1, TSC2*	25%	40%
MAP/ERK	*KRAS, MYC, BRAF, EGFR, MAP2K1, MAP3K1, NRAS*	35%	55%
DNA Repair	*MSH6, BRCA1, BRCA2, BAP1, ATM, MLH1, MSH2*	16%	45%
Chromatin Modification	*BAP1, ARID1A, PBRM1*	33%	15%

Potential associations between the mutations with PFS and overall survival OS were explored in this study. Multivariate regression analyses for PFS and OS are described in Table 4 and Table 5 respectively. In intrahepatic CCA, PFS was significantly associated with the clinical stage, *KRAS* mutations, and aberrations in the MAP/ERK pathway. The median PFS for patients with and without *KRAS* mutations was 3.4 and 10 months (p = 0.01). Median PFS for patients with and without aberrant MAP/ERK pathway genes was 3.9 and 10 months (p = 0.004). PFS was not significantly associated with age, gender, ethnicity, or presence of other mutations including *IDH1*.

**Table 4:** Multivariate regression analysis for progression free survival (PFS) in patients with intrahepatic CCA.

RISK FACTOR	REGRESSION COEFFICIENT	HAZARD RATIO (HR)	p-value	95% CI for HR
Stage III	0.7417	2.0995	0.3531	0.4388–10.046
Stage IV	1.4668	4.3352	0.0593	0.9441–19.908
KRAS	0.3110	1.3648	0.5692	0.4678–3.982
MAP/ERK	0.6120	1.8441	0.2339	0.6732–5.052

**Table 5:** Multivariate regression analysis for overall survival (OS) in patients with intrahepatic CCA.

RISK FACTOR	REGRESSION COEFFICIENT	HAZARD RATIO (HR)	p-value	95% CI for HR
Surgery	−1.248	0.287	0.031825	0.09191–0.8972
Male gender	1.177	3.25	0.010583	1.31606–8.0071
TP53	1.593	4.92	0.000645	1.97007–12.2914
KRAS	0.852	2.34	0.052791	0.98979–5.5519
FGF	−18.842	6.56e-09	0.997732	N/A

**Figure 4:**
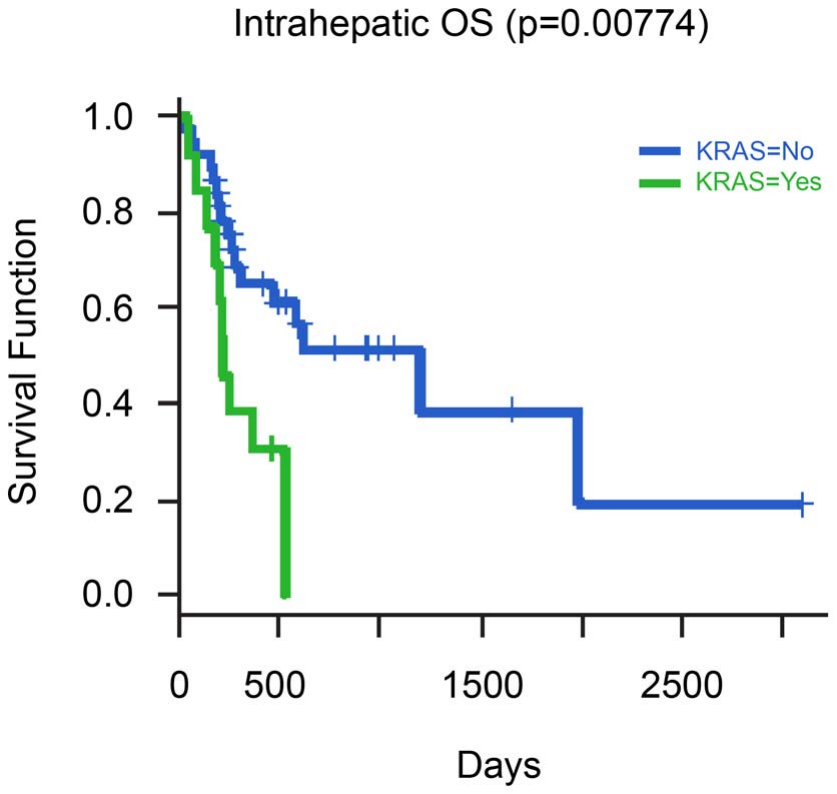
Relationship of Overall Survival with presence of *KRAS* mutation in cases of Intrahepatic CCA.

**Figure 5:**
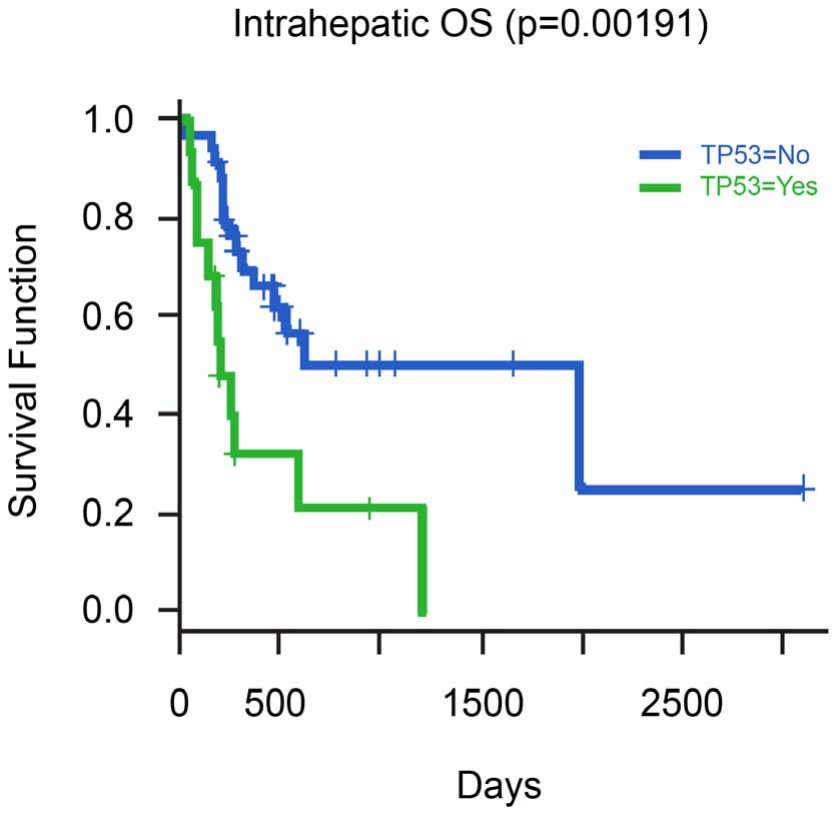
Relationship of Overall Survival with presence of *TP53* mutation in cases of Intrahepatic CCA.

**Figure 6:**
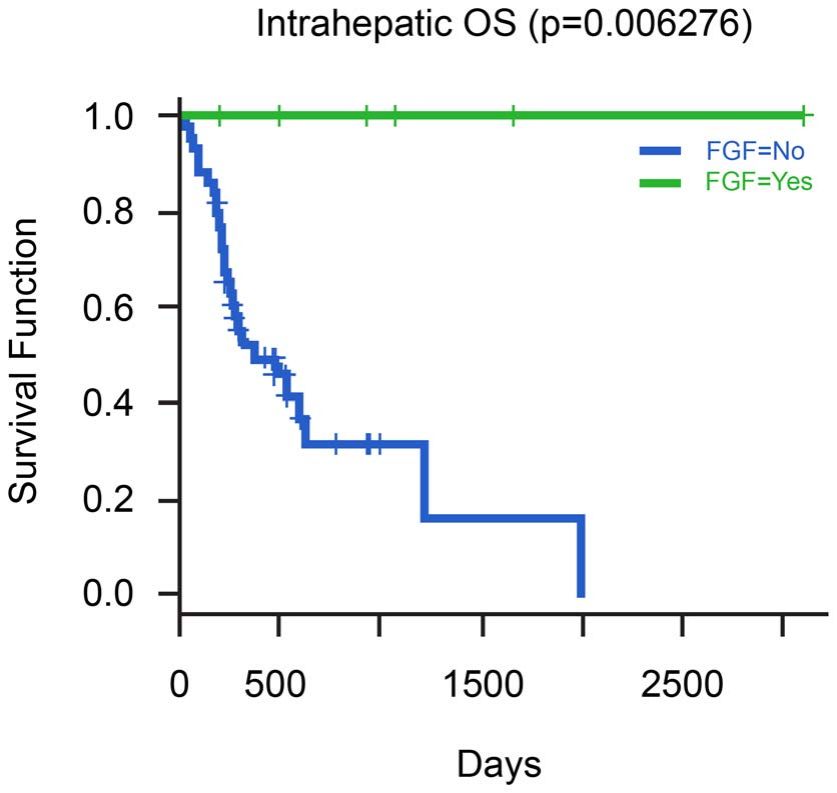
Relationship of Overall Survival with presence of *FGF/FGFR* alterations in cases of Intrahepatic CCA.

The median OS for stage II, III and IV was 20.5, 15.6 and 6 months respectively (p = 0.017). OS was significantly associated with clinical stage, surgery, gender, *TP53* mutations, *KRAS* mutations, MAP/ERK, mTOR and FGF pathway GAs (Table 6). Kaplan-Meier curves showing the relationship between OS and GA's in *KRAS, TP53* and *FGF* genes are depicted in Figs. 4, 5, and 6, respectively. A multivariate model was set up to investigate the effect of the clinical variables and GAs on PFS and OS. No factor was found to be significantly associated with PFS on multivariate analysis. However, male gender (HR 3.25, p = 0.01) and *TP53* mutations (HR 4.92, p = 0.0006) were found to be associated with reduced OS.

**Table 6:** Univariate Analysis of Survival – Intrahepatic CCA.

RISK FACTOR	MEDIAN PFS (Months)	p-value	95% CI	MEDIAN OS (Months)	p-value	95% CI
Clinical Stage						
Stage II	20.5	0.0172	4.0 – NA	NA	0.01868	NA – NA
Stage III	15.6		3.8 – NA	40.2		9.4 – NA
Stage IV	6.0		3.9–10.1	12.3		8.1 - NA
Surgery						
No	7.3	0.9968	5.0 – NA	12.3	0.0291	8.1 – NA
Yes	6.0		3.9 - NA	NA		19.5 - NA
Radiation						
No	7.1	0.6752	4.9 – NA	19.5		10.4 – NA
Yes	6.3		3.0 – NA	40.2	0.51	7.4 - NA
Gender						
Female	10.1	0.09115	5.0–20.5	40.2	0.00329	20.7 – NA
Male	4.9		3.2 – NA	7.4		6.8 - NA
Ethnicity						
Asian	5.3	0.7377	4.6 – NA	9.0	0.9659	9.0 – NA
Black	7.3		1.8 – NA	17.7		12.3 – NA
Hispanic	7.7		7.1 – NA	15.6		6.8 – NA
White	6.3		4.0 – 19.4	20.7		9.4 - NA
TP53						
No	7.7	0.5145	6.0–16.5	66.5	0.0005	17.6 – NA
Yes	4.9		3.1 – NA	6.6		4.6 - NA
KRAS						
No	10.1	0.01032	6.1–20.5	40.2	0.002588	19.5 – NA
Yes	3.4		3.0 – NA	7.4		6.1 – NA
ARID1A						
No	7.1	0.5642	4.2–16.5	19.5	0.7588	9.4 – NA
Yes	14.6		5.0 – NA	40.2		7.3 – NA
ERBB2						
No	7.1	0.4102	4.6–15.6	20.7	0.09627	12.3 – NA
Yes	NA		NA – NA	6.6		NA – NA
PBRM1						
No	7.7	0.1523	4.9–16.5	20.7	0.4427	12.3 – NA
Yes	4.8		3.3 – NA	9.4		9.0 – NA
BAP1						
No	6.3	0.363	2.9–9.7	20.7	0.746	2.7 – 38.7
Yes	7.1		0–15.2	10.4		4.3–16.5
FBXW7						
No	7.3		5.0–16.5	20.7		12.3 – NA
Yes	3.8		3.0 – NA	6.1		4.6 – NA
SMAD4						
No	7.1		4.9–15.6	20.7		12.3 – NA
Yes	3.8		NA – NA	3.2		1.7 – NA
MAP/ERK Pathway						
No	10.1	0.003578	6.3–25.6	40.2	0.01961	19.5 – NA
Yes	3.9		3.0–14.0	8.6		6.8 – NA
mTOR Pathway						
No	7.7	0.4728	5.0–19.4	66.5	0.0373	12.3 – NA
Yes	4.6		3.8 – NA	12.1		7.3 – NA
DNA Repair Pathway						
No	7.7	0.741	4.6–16.5	20.7	0.527	12.3 – NA
Yes	6.1		3.3 – NA	10.4		9.4 – NA
FGF Pathway						
No	3.1	0.2031	4.0–16.5	17.6	0.00973	9.0 – NA
Yes	14.0		6.3 – NA	NA		NA – NA
Chromatin modification Pathway						
No	7.3	0.5492	4.2–19.4	19.5	0.8472	8.6 – NA
Yes	6.1		3.3 – NA	40.2		9.4 – NA

### Extrahepatic CCA

Similar analyses were conducted for cases of extrahepatic CCAs. Median age of the 20 patients with extrahepatic CCA was 62.5 years (range 33–80). Patient demographics for this group are also described in Table 2. The median PFS for these patients was 7.7 months and median OS was 14 months.

A total of 78 GAs were identified from 20 extrahepatic CCA patient samples with an average of 3.9 GAs/patient (range 0–10). GAs identified were mutations (77%), amplifications (6%), loss/deletions (10%) and splices (6%). Only 1 tumor showed no detectable GA. Most frequent GAs were *TP53* (45%), *KRAS* (40%), *ERBB2* (25%), *SMAD4* (25%), *FBXW7* (15%), *CDKN2A* (15%) and *CDKN2B* (15%). *ERBB2* GAs included 4 mutations and 1 case of amplification. One of the *ERBB2* mutations was in the kinase domain (V777L) while 3 involved the extracellular domain (S310F). IHC for ERBB2 was negative in all cases with *ERBB2* mutations. Signaling pathways associated with mutations in extrahepatic CCAs are depicted in Table 3. MAPK, mTOR and DNA repair pathway aberrations were relatively common (55%, 40% and 45%, respectively) followed by chromatin modification (15%) and FGF signaling (5%).

**Figure 7:**
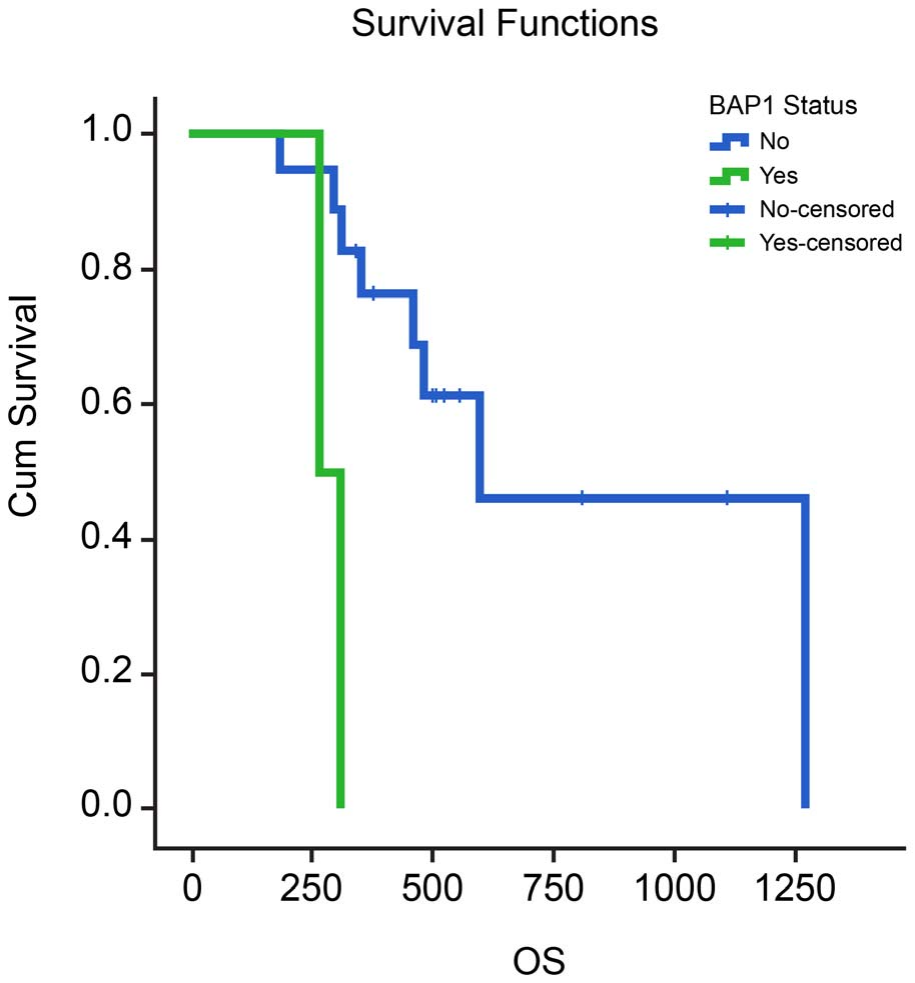
Relationship of Overall Survival with presence of *BAP1* mutation in cases of Extrahepatic CCA (p = 0.007).

Survival analysis of extrahepatic CCA cases noted significant correlation of PFS with surgery, radiation, *BAP1* mutation, and FGF pathway aberrations. OS was found to be associated with clinical stage, surgery, radiation therapy, and mutations in *PBRM1* and *BAP1* genes (Table 7). A Kaplan-Meier curve demonstrating the relationship between *BAP1* mutations and overall survival are shown in Fig. 7. The presence of *BAP1* mutations was significantly associated with reduced PFS (median: 3 vs. 8.8 months, p = 0.02) and reduced OS (median: 8.9 vs. 19.9 months, p = 0.007).

**Table 7:** Univariate Analysis of Survival – Extrahepatic CCA.

RISK FACTOR	MEDIAN PFS (Months)	p-value	95% CI	MEDIAN OS (Months)	p-value	95% CI
Clinical Stage						
Stage II	NA		10.5 – NA	NA	0.02864	NA – NA
Stage III	8.6		3.3 – NA	19.9		16.1 – NA
Stage IV	6.1	0.2516	5.7–10.1	10.4		9.9 - NA
Surgery						
No	5.9	0.0055	3.1 – NA	10.4	0.0064	9.9 – NA
Yes	40.0		8.8 - NA	42.5		16.1 - NA
Radiation						
No	6.1	0.0496	3.3 – NA	15.4	0.006144	10.4 – NA
Yes	40.0		8.6 - NA	42.5		NA - NA
Gender						
Female	6.0	0.9625	3.3 – NA	16.1	0.5226	10.4 – NA
Male	8.8		6.1 - NA	19.9		15.4 - NA
Ethnicity						
Asian	6.1	0.4504	NA – NA	NA	0.5673	NA – NA
White	8.8		6.0 – NA	16.1		11.8 – NA
TP53						
No	6.1	0.8454	3.3 – NA	16.1	0.7181	10.4 – NA
Yes	8.8		6.7 – NA	19.9		15.4 – NA
KRAS						
No	5.9	0.1864	3.1 – NA	11.8	0.213	10.4 – NA
Yes	10.5		8.8 – NA	19.9		15.4 - NA
ARID1A						
No	8.6	0.2948	6.0 – NA	16.1	0.5134	11.8 – NA
Yes	NA		NA – NA	NA		NA – NA
ERBB2						
No	8.6	0.7539	6.0 – NA	42.5	0.1248	15.4 – NA
Yes	8.8		3.1 – NA	11.8		8.9 – NA
PBRM1						
No	8.8	0.07583	6.1 – NA	19.9	0.004671	15.4 – NA
Yes	3.1		NA – NA	8.9		NA – NA
BAP1						
No	8.8	0.002	4.1–13.4	19.9	0.007	6.1–33.7
Yes	3.0		NA – NA	8.9		NA – NA
FBXW7						
No	6.7		5.3 – NA	16.1		10.4 – NA
Yes	NA		8.6 – NA	NA		NA – NA
SMAD4						
No	6.1		3.3 –NA	19.9		11.8 – NA
Yes	NA		6.7 – NA	NA		15.4 – NA
MAP/ERK Pathway						
No	3.3	0.6695	3.3 – NA	16.1	0.5933	10.4 – NA
Yes	8.8		6.7 – NA	19.9		15.4 – NA
mTOR Pathway						
No	6.7	0.3786	3.3–19.4	16.1	0.3686	11.8 – NA
Yes	24.3		6.0 – NA	42.5		10.4 – NA
DNA Repair Pathway						
No	8.6	0.5778	6.0 – NA	16.1	0.5135	11.8 – NA
Yes	8.7		3.1 – NA	19.9		8.9 – NA
FGF Pathway						
No	8.8	0.000013	6.1 – NA	19.9	0.3038	15.4 – NA
Yes	2.5		NA – NA	11.8		NA – NA
Chromatin modification Pathway						
No	8.8	0.5118	6.1 – NA	19.9	0.1392	15.4 – NA
Yes	3.1		3.0 – NA	10.4		8.9 – NA

### *BAP1* Mutations

**Figure 8:**
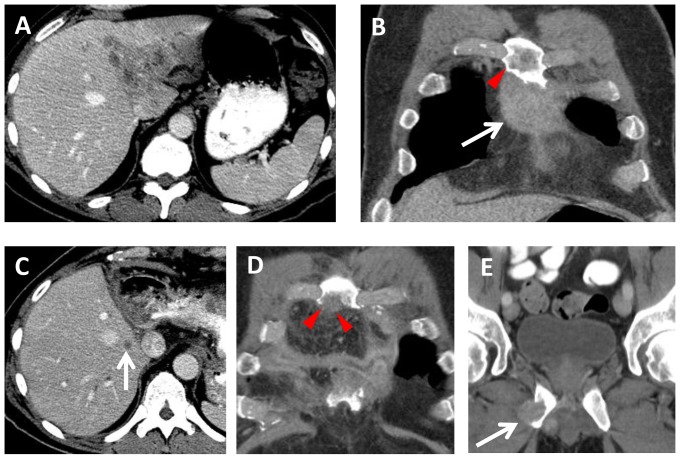
A 57 year old male with BAP1 mutated intrahepatic CCA. Baseline (A) axial and (B) coronal contrast-enhanced CT images demonstrate (A) a 6.9×5.3 cm liver mass involving segments I, II, and IV, and (B) a 5.6 cm sternal metastasis (arrow). Note the sternal metastasis does not extend to the level of the right 3rd rib (arrowhead). Extended left hepatectomy and resection of the sternal metastasis was performed. (C, D) Seven weeks later, contrast-enhanced CT images show a new metastasis in the remnant liver (arrow), and a new 2 cm recurrence (arrowheads) involving the sternum adjacent to the right 3rd rib. (E) Three months later, there is a new 2.2 cm metastasis (arrow) involving the right inferior pubic ramus.

In our series, 6 cases had *BAP1* mutations; 3 of these experienced early recurrence (less than six months) after potentially curative surgical resection. Bone and soft tissue metastases were common in this subpopulation. One such case is illustrated in Fig. 8; this patient experienced disease recurrence 7 weeks after surgical resection. In addition, all cases with *BAP1* mutation were treated with systemic chemotherapy consisting of gemcitabine and cisplatin. No radiologic response was noted and progression after first line chemotherapy occurred in all cases within a short time frame (less than 4 months). Despite the limited number of cases, the aggressive clinical prognosis of this subgroup was very evident.

### FGFR Pathway

Patients with FGFR pathway GAs appeared to have a relatively indolent prognosis in our series. FGFR Pathway GAs included mutations, fusion genes and ligand amplifications. Three patients in this subgroup had PFS>60 weeks with first line chemotherapy. These patients had *FGFR2* mutation, *FGFR2-PARK2* fusion and FGF19 amplification, respectively. Given the favorable prognosis of these cases, one patient with sustained response to systemic chemotherapy lasting for >2 years, received orthotopic liver transplantation with curative intent. In contrast, one patient with concomitant *FGFR2-NOL4* fusion had a rapidly progressive course; this patient also had co-existing *BAP1* mutation.

### Response to Targeted Therapy

**Figure 9:**
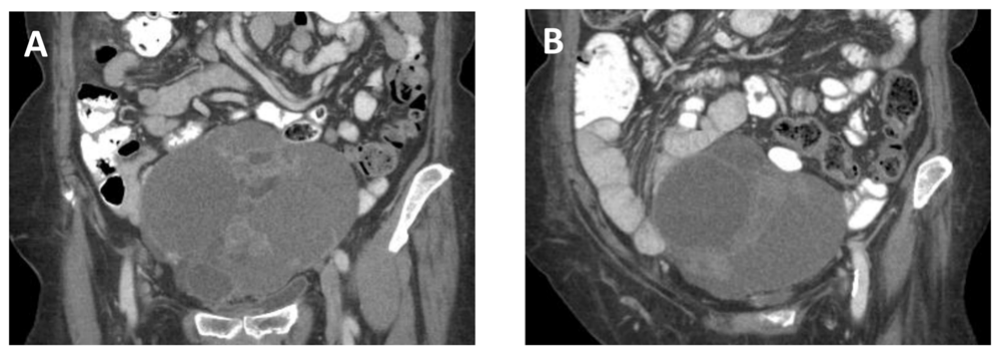
A 69 year old female with KRAS mutated CCA metastatic to the ovaries. (A) Coronal contrast-enhanced CT image demonstrates a 15.4×12.6×12.2 cm (transverse × craniocaudal × AP) pelvic mass. (B) After two cycles of trametinib + pazopanib therapy, the pelvic mass is slightly decreased to 13.5×11.3×13.4 cm.

**Figure 10:**
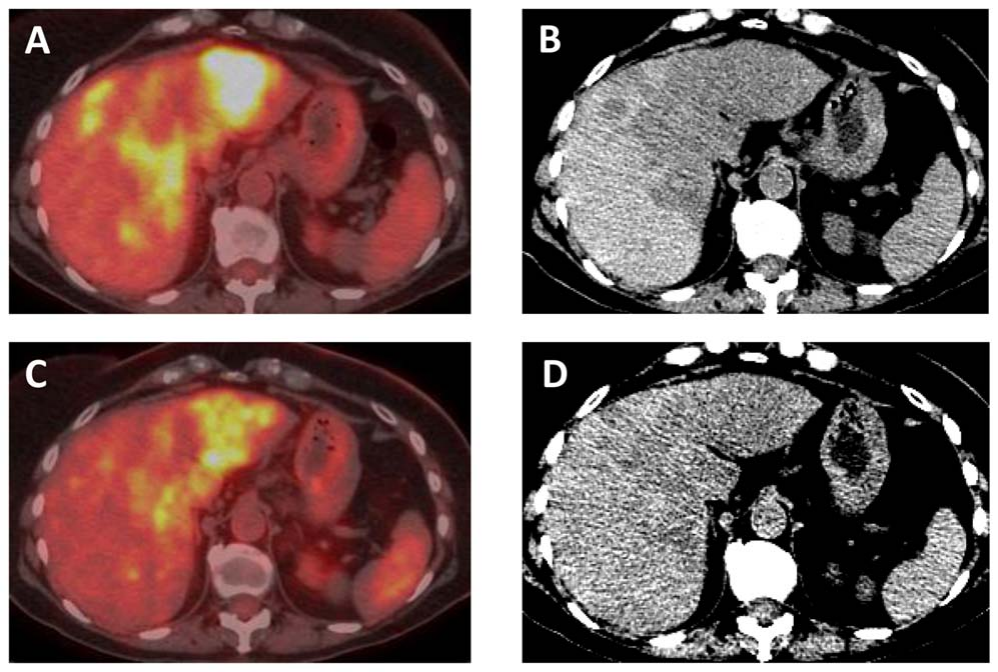
A 68 year old female with *KRAS wt* intrahepatic CCA. Axial (A) fused PET-CT and (B) unenhanced CT images demonstrate multiple confluent FDG avid liver metastases. After 3 months of erlotinib therapy, (C) axial fused PET-CT and (D) unenhanced CT images show decreased FDG avidity and slightly decreased size of metastases.

**Figure 11:**
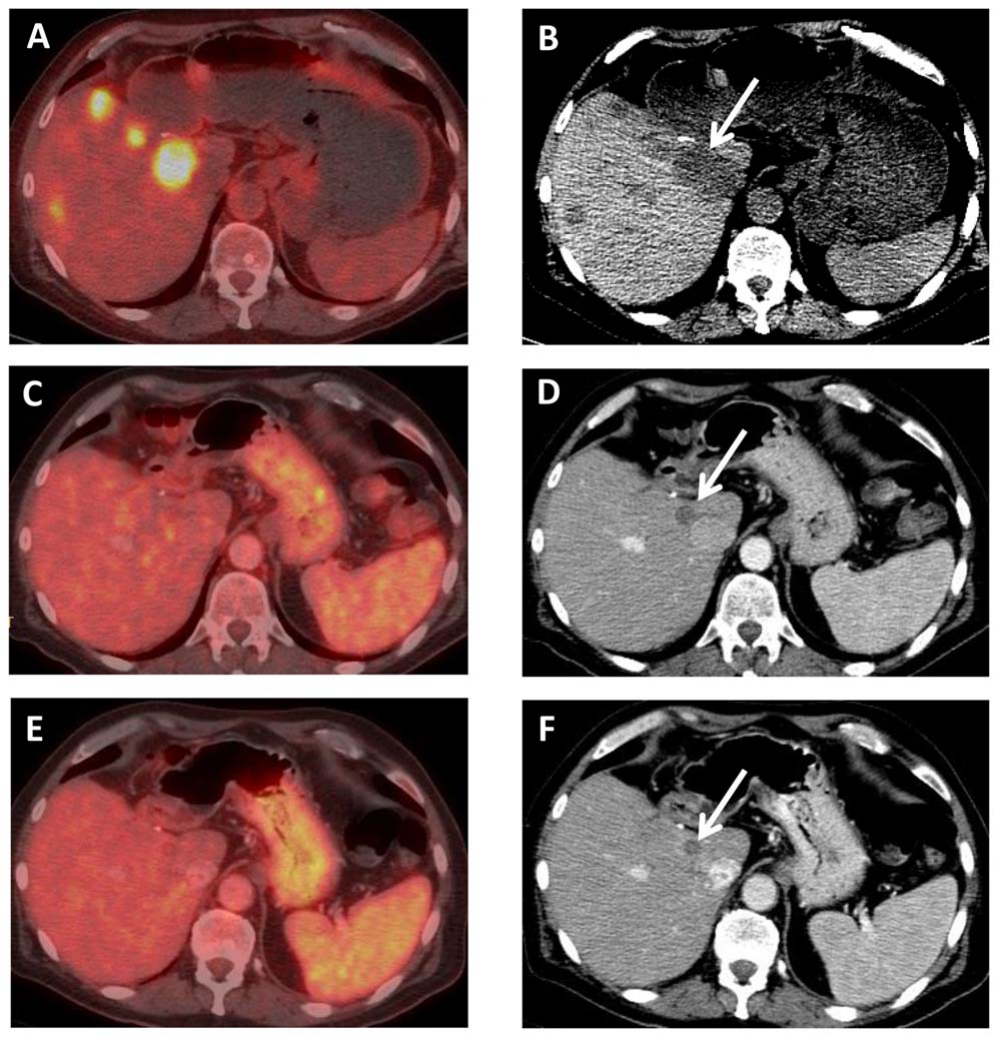
A 67 year old male with *BRAF* mutated intrahepatic CCA, who had progressed on conventional chemotherapy. Axial (A) fused PET-CT and (B) unenhanced CT images from a PET scan demonstrate FDG avidity of multiple liver metastases. After 8 weeks of BRAF inhibitor therapy, axial (C) fused PET-CT and (D) contrast-enhanced CT images demonstrate lack of FDG avidity and decreased size of liver metastases, e.g., the dominant lesion adjacent to the IVC (arrow) decreased from 3.7 cm to 1.6 cm. After 16 weeks of therapy, axial (E) fused PET-CT and (F) contrast-enhanced CT images demonstrate continued lack of FDG avidity and further decreased size of liver metastases, e.g., the dominant lesion (arrow) now measures 1.3 cm.

**Figure 12:**
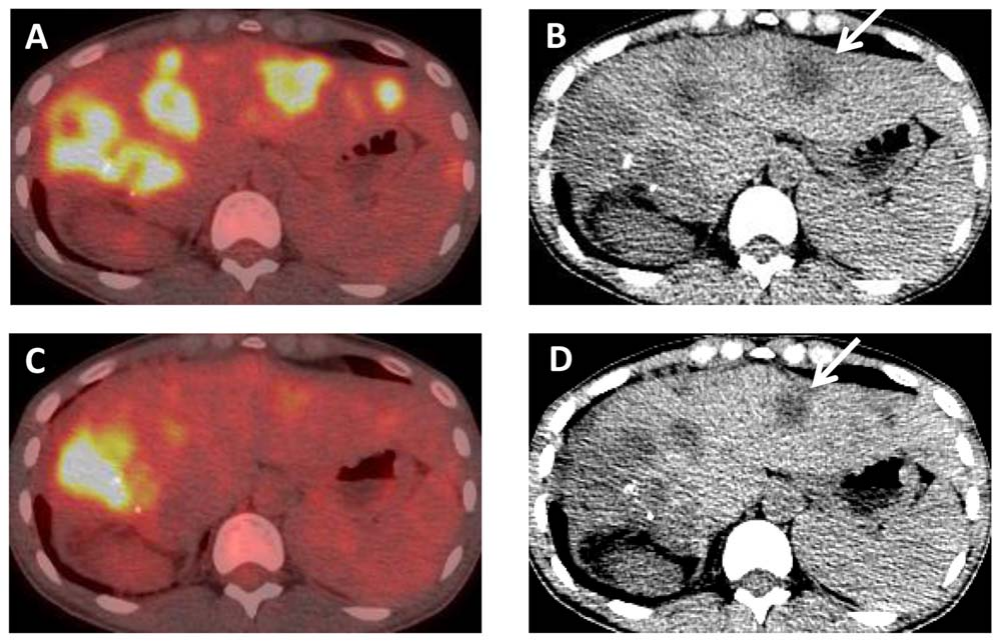
A 37 year old female with *c-MET* amplified CCA, who had progressed on conventional chemotherapy. At baseline, axial (A) fused PET-CT and (B) unenhanced CT images demonstrate multiple FDG avid liver metastases, in this patient who is status post right hepatectomy. After 4 weeks of therapy, (C) axial fused PET-CT and (D) unenhanced CT images show decreased FDG avidity and size of metastases. A representative segment II mass (arrows) has decreased from 2.6 cm to 2.1 cm.

Based on their mutational profiles, patients were referred to appropriate clinical trials. Twelve patients were enrolled in phase I or II clinical trials. Clinical outcome from targeted therapy is illustrated below: Sustained stable disease was observed in 7 cases with *KRAS* wt on erlotinib. Two cases with sustained stable disease are illustrated in Fig. 9. One patient with *KRAS* mutation was enrolled on a clinical trial with pazopanib + trametinib and experienced stable disease after prior progression on first-line chemotherapy as illustrated in Fig. 10. Two patients with *BRAF* mutation were referred for a trial with BRAF inhibitor; both experienced partial responses. One of these cases with a metabolic response on FDG-PET Scan is illustrated in Fig. 11; this patient continues on therapy for 5+ months at this time. Another patient with c-met mutation was enrolled on an expansion cohort with a c-met inhibitor and experienced a metabolic response as illustrated in Fig. 12. In case of *her2/neu* mutations, no response was observed in two cases treated with trastuzumab and lapatinib, respectively.

These cases illustrate that the mutational profile not only provided prognostic information but also provided therapeutic options.

## Discussion

Recent technical advances in genomic analysis along with reduced costs of sequencing are leading to a paradigm shift in cancer management. Several recent successes of genomic directed therapies for ‘chemo-refractory’ malignancies like melanoma, renal cancer and in subgroups of non-small cell lung cancer have further incentivized research in this field. Our goal in this study was to explore the genomic landscape of CCA, an area of unmet need in oncology with a view towards identifying disease subsets that are amenable for targeted therapy. In this study, we chose NGS platforms that can identify clinically ‘actionable’ GAs from FFPE tumor specimens. The use of FFPE samples for obtaining reliable NGS data is technically challenging but has the potential of rapidly bringing the vast scope of this technology to the clinical setting. In this study, we explored a) genetic differences between intrahepatic and extrahepatic CCA, b) the prognostic importance of the GAs and c) the impact of the targeted therapy.

As noted earlier, there have been several recent exome-sequencing studies of intrahepatic CCA. There are however very limited data regarding the mutational spectrum of extrahepatic CCA and although the patient numbers in this subgroup are limited, interesting differences are highlighted by this study. Higher frequency of *KRAS, P16* and *SMAD4* mutations in extrahepatic CCA suggest a molecular phenotype that resembles pancreatic cancer rather than intrahepatic CCA. *IDH1* and *ERBB2 GAs* occur exclusively in intra and extrahepatic CCA, respectively. These results contrast with the GAs seen in gallbladder cancer, where ERBB2 amplification (not mutation) occurs in 15% of the cases.

The prognostic importance of these GAs is highlighted by the association between survival and *BAP1* mutation. Jiao et al., noted frequent mutations of chromatin-modulation genes including *BAP1, PBRM1* and *ARID1A* and an adverse prognosis was noted in the *BAP1* mutated cases [11]. In our study, 25 cases (33%) had GAs involving at least one of the chromatin modulating genes. These genes may behave as tumor suppressors and are frequently mutated in solid tumors. BAP1 (BRCA1-Associated Protein 1) was initially identified as a protein that binds to BRCA1 and is now recognized as a tumor suppressor that mediates its effects through chromatin modulation, ubiquitin-proteasome system and the DNA damage response pathway. Germline *BAP1* mutations confer susceptibility to uveal melanoma, epithelioid atypical Spitz tumors, cutaneous melanoma, and mesothelioma [17]. Somatic *BAP1* mutations are infrequent but have been reported in prostate, ovarian, colon, breast, lung cancers and in mesothelioma [18]. *BAP1* loss is associated with an aggressive metastatic phenotype in uveal melanoma and in renal cancer [19]. BAP1 loss in uveal melanoma predicts increased risk of liver metastases after enucleation surgery, thereby highlighting the need for careful patient selection for this radical surgical procedure [20]. Interestingly, our study also noted early recurrence after surgery in the presence of *BAP1* mutation. We propose that surgically resected CCA cases with *BAP1* mutation be considered for neoadjuvant approaches, so that their disease biology can be identified before considered major hepatic resection. Several small molecule inhibitors have been developed against chromatin regulators; three (HDAC, JAK2 and DNMT inhibitors) are already approved by the Food and Drug Administration (FDA) [21]. The role of these inhibitors in CCA deserves further investigation.

Other negative prognosticators in the study included the presence of *KRAS* mutation. Chen et al., had previously reported a poor prognosis and presence of peri-neural invasion in the presence of *KRAS* mutation in CCA [22]. One case in our series with mutated *KRAS* experienced disease stability after prior progression on chemotherapy with trametinib + pazopanib. Response to MEK inhibitors in this setting has also been reported by other studies and this option may be worth investigating further for *KRAS* mutated CCA [23]–[25].

The presence of *ERBB2* mutations also contrasts with the GAs reported in gallbladder cancer, wherein *ERBB2* amplifications have been reported [26], [27]. *ERBB2* GAs occurred in 6 cases, which included 2 amplifications and 4 somatic mutations. *ERBB2* mutations have been described in non-small cell lung cancer, breast cancer, gastric cancer and colorectal cancer [28], [29]. These mutations have not been described to our knowledge in CCA. Unfortunately, these mutations may be more difficult to target than *ERBB2* amplifications [30]. Yoshikawa et al., measured ERBB2 expression in 236 cases of CCA by immunohistochemistry (IHC). They reported a 0.9% and 8.5% positive ERBB2 expression rate in intrahepatic and extrahepatic CCAs, respectively [31]. In our cases with *ERBB2* mutation, IHC expression of ERBB2 was not detectable in our study; one of the *ERBB2* mutations was in the kinase domain (V777L) while 3 involved the extracellular domain (S310F). Based on the current knowledge of *ERBB2* biology, trastuzumab or lapatinib therapy may be value in the extracellular domain mutations but could be of limited clinical benefit for kinase domain mutations and no response to either agents was noted in our cases. Bose *et al.*, noted that V777L mutation was associated with negative ERBB2 protein expression and resistance to lapatinib [32]. Irreversible small molecule tyrosine kinase inhibitors (TKIs), including afatinib, neratinib and dacomatinib may be of value in this setting [33]. Furthermore, since *ERBB2* activation can transactivate several other signaling mechanisms, combinations of *ERBB2* directed TKIs with trastuzumab, mTOR or MEK inhibitors may be of value in this disease given its genomic profile.

GAs in the FGFR pathway were noted in 9% of our cases, most representing *FGFR* mutations. Three cases had *FGFR2* fusion genes; these have been described before in CCA and are oncogenic *in vitro* [34]. Preclinical data indicate that these fusion proteins may indicate tumor susceptibility to targeted FGFR inhibitors, BGJ398 and PD173074. Recent data from Arai *et al.*, indicate that *FGFR2* fusion genes occur in 13.6% of intrahepatic CCA cases; Borad et al. have described responses to targeted *FGFR* inhibitors in this patient subgroup [35], [36]. This was also noted in our patient with *FGFR* fusion. The relatively good prognosis of this molecular subset is intriguing and contrasts with the poor survival of the *p53* mutated subtype. Interestingly, similar findings were reported recently in bladder cancer, wherein whole genome transcriptome profiling revealed three molecular subtypes with differing prognoses [37]. The p53-subtype was resistant to chemotherapy while cases with *FGFR* mutation constituted a luminal subtype with good prognosis. The superior survivals of the FGFR mutated CCA subtype, if confirmed may potentially stratify these cases for liver directed therapy. One case in our series with FGFR mutation and prolonged disease stability received liver transplantation.

Other mutations identified in our study include *BRAF* and *C-MET*, both of which were associated with impressive responses seen with targeted therapy. The incidence of these mutations in CCA is low (estimated at 3% for *BRAF V600E* in intrahepatic CCA)[38] but the sustained responses seen with BRAF inhibitor deserve further exploration. *IDH1 (R132C)* mutations represented a significant number of cases that may be potentially targetable, given the advent of potent IDH inhibitors [39]–[44].

The IPA suggests genomic homology between subsets of CCA with glioblastoma, melanoma and certain categories of non-small cell lung cancer, thereby supporting the role for molecular rather than morphologic classification of human cancers. A limitation of our series is the relatively small number of cases of extrahepatic CCA and those treated on therapeutic clinical trials. Unfortunately, clinical trials are lacking for this orphan disease population.

We recognize however that the detection of candidate mutations does not necessarily indicate its relevance as a prognostic biomarker or a potential therapeutic target. An integrated approach that includes functional genomics is required to leverage the full potential of NGS and realize the promise of precision medicine. In conclusion, genomic sequencing can potentially identify distinct molecular subsets of CCA, with distinct prognostic and therapeutic implications.

## Supporting Information

S1 Table **Detailed results of GA's including gene affected, type of alteration, allele frequency or copy number.**
(XLSX)
